# Hepatitis B Virus Antibody Levels in High-Risk Health Care Workers

**DOI:** 10.5812/kowsar.1735143X.707

**Published:** 2011-08-01

**Authors:** Esmaeil Mohammad Nejad, Sirous Jafari, Mahmood Mahmoodi, Jamaloddin Begjani, Seyyedeh Roghayyeh Ehsani, Narmela Rabirad

**Affiliations:** 1 Nursing Department, Islamic Azad University, Aliabad Katoul Branch, Aliabad Katoul, IR Iran; 2 Department of Infectious and Tropical Diseases, School of Medicine, Tehran University of Medical Sciences, Tehran, IR Iran; 3 Epidemiology and Biostatistics Department, School of Public Health, Tehran University of Medical Sciences, Tehran, IR Iran; 4 Nursing Department, Islamic Azad University, Iranshahr Branch, Iranshahr, IR Iran; 5 Imam Khomeini Clinical and Hospital Complex, Tehran University of Medical Sciences, Tehran, IR Iran; 6 Secretary of Infection Control Comittee, Tehran University of Medical Sciences, Tehran, IR Iran

**Keywords:** Hepatitis B virus, Health care providers, Vaccination

Dear Editor,

Hepatitis B is one of the most prevalent infectious diseases in the world, with more than two billion people infected and more than 350 million chronic carriers [1][2][3][4]. Approximately five hundred thousand infected patients die annually [5]. Health care workers are also at high risk of contracting Hepatitis B virus (HBV) through blood, and infected staff can transfer HBV to uninfected patients, which can further spread the infection into society [6]. In addition to standard precautions, personnel who are sensitive and susceptible or infected should be identified and immunized to reduce the morbidity of health care workers [7]. Health care workers, particularly those working in emergency departments, are considered to be a high-risk group [8][9]. We conducted this descriptive, cross-sectional study to determine the HBV antibody titer and immunity levels of health care workers and 173 personnel working in an emergency department. We evaluated subjects' HBS antibody titer with an ELISA method. Titer < 10 IU/L, titer ≥ 10 to 100 IU/L, and titer > 100 IU/L were considered to be inappropriate, protective, and good levels of immunity, respectively. With these cutoffs, 31 (17.19%), 72 (61.41%), and 60 (68.34%) of subjects had inadequate, protective, and good antibody titer levels, respectively.

Additionally, 84.97% of all subjects had been completely vaccinated against Hepatitis B, and 14 (91.83%) were not immunized against Hepatitis B. The patients in this latter group (the nonimmunized group) were statistically similar to patients with incomplete vaccinations in terms of antibody titer level (P < 0.002).This measurements were 81%, 88.1%, and 35% in Goldberg et al., Room et al., and Barash's studies, respectively [10][11][12], and the figures in other studies in Iran were 86.4% and 96.3% in Khosravi et al. in Fars Province and Allami et al. in Qazvine, respectively [13][14]. Our results showed a significant relationship between age and hepatitis B antibody level similar to the level noted in Bonanni et al. in Italy. Our finding is not consistent with Shein et al.'s finding in an American sample or the level found in Fonderberg et al. [6][15][16]. It seems that the immune response in adults is reduced as a result of conditions induced from changes dependent on age, such as inadequate supplies of blood, malnutrition, drugs, and metabolic changes [17]. Our research shows that smoking reduces the immune response to the hepatitis B vaccine. We also found that smoking was one of the significant factors associated with vaccination failure response. This finding is similar to the findings by Tolosa et al. in a Spanish sample and Adverhoff et al. but is inconsistent with Barash et al.'s finding in an American sample [12][18][19]. Because antibody levels in the complete vaccination group were higher than in the incomplete vaccination group, hospital authorities should screen incomplete vaccination cases and encourage them to complete the vaccination series and assess ways to promote immunity levels in high-risk groups. Although most resources have suggested that the production of immune antibody titer after all three HBV vaccination is about 95% [7], one month after the last injection, immunity status should be evaluated. In previous studies, repeating all three vaccinations in subjects with an inappropriate response induced immune induction in about 50% of cases [12][14].Vaccination against hepatitis B in health care workers, especially in those with titers fewer than 10 IU/L, is essential. Furthermore, assessing patients> immunological response to vaccines is needed in the years after vaccination, and continued vaccination against HBV by way of an extensive immunization program is the best way to control HBV infection.
